# Values and preferences of female sex workers in Zimbabwe for long-acting injectable pre-exposure prophylaxis and the dapivirine vaginal ring: results of a mixed-methods research study

**DOI:** 10.1136/bmjgh-2025-021333

**Published:** 2026-03-02

**Authors:** Fortunate Machingura, Marc d'Elbée, Tatenda Kujeke, Jasper Maguma, Michelle Rodolph, Rachel Baggaley, Sungai T Chabata, Frances M Cowan

**Affiliations:** 1 Centre for Sexual Health and HIV AIDS Research (CeSHHAR) Zimbabwe, Harare, Zimbabwe; 2 Department of International Public Health, Liverpool School of Tropical Medicine, Liverpool, UK; 3 National Institute for Health and Medical Research (Inserm), Research Institute for Sustainable Development, University of Bordeaux, Bordeaux, France; 4 Global HIV, Hepatitis and STI Programme, World Health Organization, Geneva, Switzerland; 5 World Health Organization, Geneva, Switzerland

**Keywords:** AIDS, Africa, Zimbabwe, Other study design

## Abstract

**Introduction:**

Female sex workers (FSWs) in sub-Saharan Africa are at high risk of HIV acquisition. Here we explore the values and preferences of Zimbabwean FSW for long-acting pre-exposure prophylaxis (PrEP).

**Methods:**

We employed mixed methods; focus group discussions (FGD) (n=15), a respondent-driven sampling (RDS) survey (n=4444) from 22 sites across Zimbabwe and a nested discrete choice experiment (DCE) (n=435) conducted in 4/22 sites in 2021. Purposively selected FSWs aged 18 or over who reported being HIV negative were eligible for inclusion in FGDs. Analysis of self-reported HIV negative survey participants was RDS-II weighted. DCE analysis estimated relative preferences. Qualitative and quantitative data were triangulated.

**Results:**

Median age of survey participants was 28 years with IQR of 23–34 years. There was strong concordance across methods by product, provider, service and individual characteristics. Most FSWs indicated that they preferred long-acting injectable (LAI) PrEP to either oral PrEP or dapivirine vaginal ring (DVR). Most were interested in using LAI PrEP (74.1%; n=1835/2392), a few the DVR (10.9%, n=230/2392), and 2.4% (59/2392) and 13.5% (268/2392) either or neither of the two options, respectively. There was little trust in public sector healthcare providers, with most FSWs opting to access PrEP through programmes designed for sex workers (and stating they would miss a prescription refill/repeat injection if the public sector was the only available option). Injectable PrEP addressed privacy and adherence concerns to some extent, although FSWs felt that 6-monthly would be preferable to less frequent injections. Issues of privacy (related to PrEP and being a FSW), confidentiality and respect emerged as key qualitative themes.

**Conclusions:**

FSW had a strong preference for LAI PrEP, but ensuring product choice and user privacy was key. FSW in Eastern and Southern Africa should therefore be prioritised for PrEP choices, with ongoing monitoring and evaluation of services to make sure they are acceptable, effective and evolve as products and delivery options become available.

**WHAT IS ALREADY KNOWN ON THIS TOPIC**

- Since 2015, the WHO has recommended daily oral pre-exposure prophylaxis (PrEP) for people at substantial risk of HIV (defined as HIV incidence greater than 3% per annum in their population group). More recently, longer-acting PrEP options, which negate the need for daily pill taking, such as the dapivirine vaginal ring (DVR) and injectable long-acting injectable PrEP, have been recommended and prequalified by WHO. Long-acting PrEP is widely preferred (over daily PrEP) in values and preferences studies among adolescent girls and young women in Africa as well as men who have sex with men, but the values and preferences have not been examined among female sex workers in Southern Africa who are at very substantial risk of acquiring infection.

**WHAT THIS STUDY ADDS**

- This mixed-method study, which combines qualitative, survey and discrete choice experiment data, found across all research methods that female sex workers had a strong preference for long-acting injectable PrEP over oral daily PrEP and the DVR. Maximising the time between injections was important, as was ensuring product choice and privacy of users.

**HOW THIS STUDY MIGHT AFFECT RESEARCH, PRACTICE OR POLICY**

- These study findings offer new perspectives on the values and preferences of southern African female sexual workers, as well as likely barriers and opportunities to support long-acting PrEP uptake. Our data suggest that having a choice of PrEP options may motivate people who would not otherwise take PrEP to consider doing so, highlighting the potential for these products to fill important gaps in effective prevention coverage.

## Introduction

 Despite the availability of effective biomedical prevention for HIV,^1^ UNAIDS global HIV prevention targets of 370 000 new infections per annum by 2025 will be missed by a wide margin. The Joint United Nations Programme on HIV/AIDS (UNAIDS) estimates that 49% of new infections in sub-Saharan Africa in 2022 were among key populations and their sexual partners^2^ (defined by UNAIDS as those people who are at higher risk of HIV and other health issues due to their behaviours); this includes female sex workers (FSWs).^3 4^ HIV incidence among FSW is nine times that of all women and five times greater in Eastern and Southern Africa.^5^ Young women who sell sex are especially vulnerable, due to relationship power imbalances related to their age and gender. Women who sell sex do so against a background of intense stigma and discrimination exacerbated by criminalisation of sex work.^6^

Since 2015, WHO has recommended daily oral pre-exposure prophylaxis (PrEP)^7^ for people at substantial risk of HIV (defined as greater than 3% per annum). 79 countries have National PrEP Guidelines in place.^8^ Despite this, effective use of oral PrEP across Africa is often poor.^9^ Qualitative and quantitative data suggest barriers to uptake and continuation, some of which relate to daily pill taking including inconvenience of carrying pills, requirement to attend health facilities for prescription refills, fear that taking oral PrEP might be misconstrued as taking ART or inadvertently expose their sexual activity to family and friends.^9^

In 2021 and 2022, large trials of injectable cabotegravir (CAB-LA), given by 2 monthly injection, demonstrated its superiority compared with daily oral emtricitabine and tenofovir disoproxil fumarate in both men^10^ and women.^11^ Since 2022, WHO has recommended that CAB-LA be offered to those at substantial risk of HIV infection as an additional prevention choice. CAB-LA is licensed for prevention in 57 countries. The monthly dapivirine vaginal ring (DVR) has also been recommended by WHO as an additional prevention choice. CAB-LA and the DVR are being scaled up to a limited extent across Africa.^10^,^13^ More recently, the randomised component of a large phase 3 trial (PURPOSE 1) of the long-acting 6-monthly subcutaneous injectable lenacapavir in African women was stopped mid 2024 following a preplanned interim analysis because there were no incident infections in the lenacapavir arm (follow-up continues), although incidence in two different daily oral PrEP arms was similar to background incidence.^14^ Long-acting injectable PrEP (LAI PrEP) and DVR negate the need for daily pill taking. In values and preferences studies,^15^ LAI PrEP is widely preferred over daily pill taking, although few studies have been conducted in African FSW. Since the release of PURPOSE1 results, things have moved quickly with lenacapavir now prequalified by WHO and licenced for use in the USA, Europe and South Africa. In September 2025, Unitaid and the Gates Foundation announced partnerships with two generic manufacturers which mean that Lenacapavir for PrEP will be available at a cost of US$40/year in low-income countries from 2027. Lenacapvir for PrEP was licenced in Zimbabwe in November 2025.^8^

Here, we report on a study to explore values and preferences for long-acting PrEP choices, including LAI PrEP and the DVR and their delivery options among FSW in Zimbabwe in 2021. The study was nested within the AMETHIST trial^16 17^ and Zimbabwe’s nationally scaled Key Populations programme for sex workers.^18 19^

## Methods

**Figure 1:**
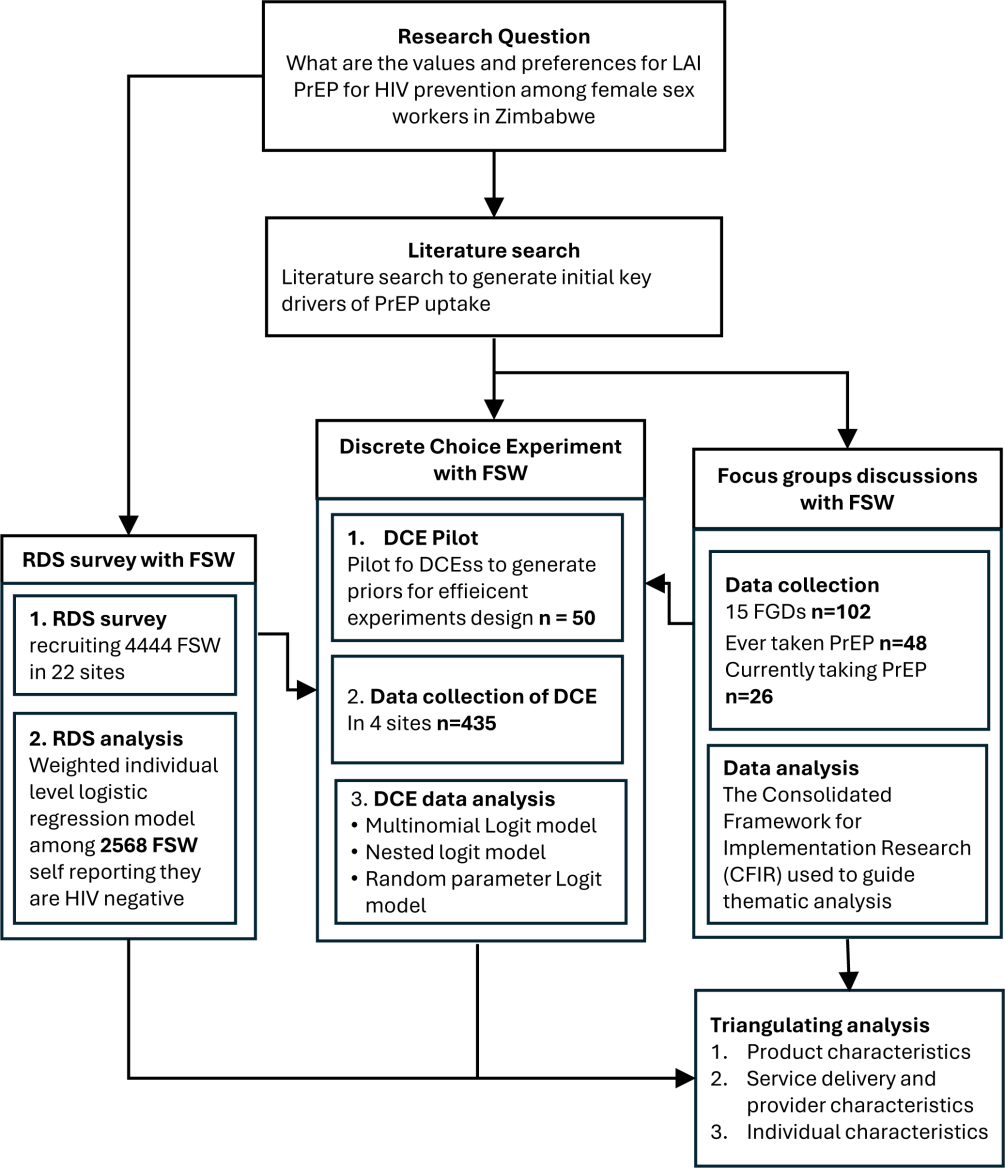
Overview of mixed research methods. DCE, discrete choice experiment; FGD, focus group discussion; FSW, female sex worker; LAI, long-acting injectable; PrEP, pre-exposure prophylaxis; RDS, respondent-driven sampling.

We employed a mixed-methods design combining focus group discussions (FGD), a respondent-driven sampling (RDS) survey and a discrete choice experiment (DCE) to identify FSW preferences for the uptake and delivery of LAI PrEP and the DVR, and to explore behaviours and perceptions that may underlie preferences. Figure 1 illustrates the research design, detailing the methods used for data collection, the data analysis process and how the methods supported and built on each other to provide a comprehensive methodological framework for understanding preferences.

We conducted FGD to explore group perceptions and previous PrEP use choices and experiences. Our DCE, a quantitative method for measuring stated preferences for goods or services, was informed by our qualitative research. In addition, we included questions about PrEP preferences in the AMETHIST trial endline survey among FSW.

### Data collection

#### Qualitative data collection

The formative qualitative study was conducted between August and December 2021 among FSW in Harare and Mutare, Zimbabwe. FGD participants (n=102) were both attendees and non-attendees of the nationally scaled programme for sex workers (The Key Populations Programme).^18 19^ To recruit non-attendees, we conducted rapid ethnographic mapping which combined identification of sex work locations with additional data on the social context in which sex is exchanged.^20^ Participants were purposively selected to include past or current PrEP users and those who had been offered but opted not to take PrEP. They were eligible for FGD participation if they were 18 years or older and self-reported being HIV negative. Basic demographic data (age, education, marital status, type of accommodation) were collected from each participant.

Fifteen participatory FGDs were conducted in venues that were both accessible and private. Participants with different PrEP use experience were allocated to different groups (FGDs n=5 non-PrEP users; n=4 past PrEP users; n=3 current PrEP users). Discussions followed a topic guide and used vignettes and role-plays. The discussion explored attitudes to PrEP choice, adherence, continuation and whether their prevention needs were currently being met. Barriers and facilitators to PrEP access were discussed. Interviewers had an actual sample of each potential PrEP modality (oral, vaginal ring and injection to view) and used these to promote discussion around acceptable forms of PrEP delivery.

#### Development of DCE attributes and levels, and the DCE questionnaire

Thematic coding produced an initial list of attributes from all FGD, which was refined over two further iterations centred on FSW’ priorities into broader themes representing final attributes and levels.

For estimating DCE parameters with the minimal required sample size, four different DCE questionnaires composed of a series of 12 scenarios were generated using a d-efficient design created in Ngene software (V.1.21.1; ChoiceMetrics, Sydney, New South Wales, Australia). DCE questionnaires were evenly distributed among the participants. Each scenario presented two alternative approaches, defined by seven attributes and their corresponding levels, for initiation and continuation of long-acting PrEP options, and an opt-out (‘neither’ option) presented as ‘I would not use a long-acting PrEP option if these were the only two options available’ (see table 1). Pictorial representations of these scenarios were developed to facilitate comprehension of the alternative services (see example in online supplemental appendix table S4).

**Table 1:** Attributes and levels for discrete choice experiment

	Domain	Attribute	Levels
1	Service delivery characteristics	Location	Government clinic, Sex worker clinic, pharmacy
2		Proximity	In my neighbourhood, outside my neighbourhood
3		Support	Assisted administration, self-administration
4	Product characteristics	Frequency	Once a month, once every 3 months
5		Fee	Free, US$1, US$3
6		Efficacy	35%, 85%, 90%
7		Mode of administration	Injection, Vaginal ring

A DCE design with neutral alternatives within scenarios (unlabelled) was preferred because there were no alternative-specific parameters in the experiment.^21^ Participants were eligible for the DCE survey if they were cisgender female, self-identifying as sex workers, ≥18 years and HIV-negative.

#### RDS and DCE data collection

The RDS survey was conducted between September and December 2021 among FSW in 22 sites around Zimbabwe as part of the AMETHIST trial endline survey.^16^ The size of the survey was determined by AMETHIST trial outcomes. The DCE was nested within the RDS survey with FSW participants drawn from four of the 22 survey sites including a border/tourist town and a tourism centre, a commercial farming area, a mining town on a major highway and a university and mining town.

The RDS survey aimed to recruit 200 FSW in each trial cluster (n=4400 overall); methods have been described in detail elsewhere.^16 17^ In brief, we conducted mapping followed by purposive selection of six sex worker ‘seeds’ per site representing a mix of ages, sex work typologies and geographic locations. Seeds self-completed an interview using audio computer-assisted self-interview (ACASI) directly onto tablet computers, had a blood sample collected for HIV testing and were given two coupons to distribute to peers. Women receiving a coupon could enrol, self-complete an interview using ACASI, provide biospecimens and were subsequently given two coupons to recruit two of their peers. Five to seven iterations of this process (‘waves’) were performed excluding the seeds. Participants were given US$5 compensation for their time, and US$2 for each referral who was eligible and recruited. We used an in-house coupon management system to track coupons and all coupons were verified and redeemed only once to avoid repeat participation.

FSWs were assessed for eligibility when they presented at the survey sites and were eligible to participate in the RDS survey if they were aged ≥18 years, currently working as a sex worker (had exchanged sex for money or goods in the past 30 days) and had been living or working in the survey site for at least the previous 1 month. The questionnaire included questions on demographics, sex work, sexual behaviour and condom use, HIV testing history, stigma, experience of violence, quality of life,^22^ mental health,^23^ alcohol use,^24^ relationships with other sex workers, engagement with sisters’ services and use of sexual and reproductive health services. In addition, the survey included four questions about long-acting PrEP (paraphrased here) (1) ….which of these new PrEP options would you be interested in taking? (2) Would 2-monthly LAI PrEP be easier for you to take than taking a daily tablet? (3) Would having a vaginal PrEP ring (changed monthly) be easier to than taking a daily tablet? (4) Would it be easier to use 2 monthly LAI PrEP or a monthly vaginal PrEP ring?, which were answered by all FSW who were tested HIV negative around the time of questionnaire completion (see online supplemental appendix table S1 for complete wording). We collected data to determine personal network size for RDS weighting.

### Qualitative data analysis

#### Data management

FGD recordings were transcribed verbatim and translated. The analysis was guided by the Consolidated Framework for Implementation Science Research (CFIR)^25^ which provides a framework to assess the barriers and facilitators that affect the success of implementing an evidence-based intervention. CFIR domains of product attributes (eg, price and delivery method), provider (eg, type of provider) and service delivery (eg, location of services) characteristics and individual characteristics emerged in the analysis and were used as a basis to organise the analysis and to identify emergent themes.

The qualitative data, while informing the DCE design, were also analysed to provide additional depth in understanding preferences. Coding frameworks were developed, and emerging themes were identified through collaborative analysis of the field notes and data and in-person discussions of 6–7 researchers. Transcripts were coded in NVivo (V.11; QSR International, Burlington, Massachusetts, USA) by one researcher, with an independent coder checking a purposive 10% of transcripts for intercoder reliability.^26 27^ Data were analysed to ensure that commonalities or differences between individuals and groups as units were visible.

A detailed description of the sociodemographic characteristics of FGD participants is provided in table 2 including information on age, education level, marital status and other relevant factors that may influence their perspectives on PrEP options.

**Table 2:** Characteristics of FSW participating in FGDs

Characteristics	FSW participating in FGDs (N=102) n (%)
Age	
18–24	39 (38.2%)
≥25	63 (61.9)
Years in sex work	
≤2 years	19 (18.6%)
> 2 years	83 (81.4%)
Marital status	
Single/never married	42 (41.1%)
Married/cohabiting	7 (6.9%)
Widowed/divorced	53 (52.0%)
Education	
None/primary	3 (11.7%)
Secondary or higher	108 (91.2%)
Typology*	
Bar based	46 (23.6%)
Street based	43 (21.9%)
Escort based	12 (6.1%)
Home based	63 (32.1%)
Venue based	12 (6.1%)
Highway based	20 (10.2%)
Attended key population programme ever	
Yes	96 (94.1%)
No	8 (7.8%)
Ever taken PrEP	
Yes	48 (47.2%)
No	54 (52.8%)
Currently taking PrEP	
Yes	26 (25.5%)
No	76 (74.5%)

* Multiple response allowed.

FGDs, focus group discussions; FSW, female sex worker; PrEP, pre-exposure prophylaxis.

#### RDS survey analysis

Analysis was reported in accordance with the STROBE-RDS guidelines.^28^ We generated recruitment trees to judge onward recruitment by FSW. We assessed whether final estimates of key outcomes converged over the six waves of recruitment and whether the social networks of FSW appeared to have been disconnected (bottlenecks) using combined convergence and bottleneck plots in each site.

To improve sample representativeness and generate unbiased estimates, all analyses were RDS-II weighted, with women’s responses weighted by the inverse of the reported number of sex workers they knew, that is, the number of other women that she could have recruited to the survey. The rationale for RDS-II weighting has been described in detail elsewhere.^29 30^ We described the sociodemographic characteristics of all HIV-negative participants by comparing data from the 18 non-DCE sites to the sample included in the DCE study, focusing on characteristics that may influence PrEP preferences.

#### DCE analysis

DCE data were cleaned using Stata Statistical Software (V.14; StataCorp). Utilities (U), representing the strength of relative preferences, were estimated using discrete choice models in Nlogit V.6 Software. DCEs assume that choices are made according to the utility maximisation principle, where the best choice provides the highest utility/satisfaction to the decision maker. We estimated prior utility parameters from a pilot study (N=45) using a multinomial logit (MNL) model. These priors informed the DCE questionnaire design in Ngene, improving experimental efficiency and our ability to capture preidentified relative preferences. For the main analysis, choice data, elicited from the choice made between the service alternatives, were first analysed using a model as a basic model. Random parameter logit and generalised mixed logit models were then introduced to respectively allow for unobserved preference heterogeneity in addition to scale heterogeneity.^21^ All attribute levels were effects coded.^31^

Key preferences elicited from the DCE and qualitative data were categorised into the following CFIR domains.^25^ Findings within each of these categories were triangulated across methods and classified as consistent, complementary (if providing more depth or a different perspective) or contradictory.

### Patient and public involvement

FSWs were involved in the design, implementation, interpretation and dissemination of study results. CeSHHAR is well known in communities and CeSHHAR’s Key Populations programme has worked across Zimbabwe since 2009. CeSHHAR has MoUs with all district councils in which it works and meets regularly with key stakeholders including political and traditional leadership and police. We conducted intensive community sensitisation prior to the study taking place. CeSHHAR was at the forefront of establishing Zimbabwe’s National Key Populations forum where this research has been presented both at early stages of planning and development and then when results were disseminated.

## Results

Characteristics of FGD participants are detailed in table 2 and the RDS and DCE in table 3 and reflect representation among FSW across age, duration in sex work, marital status, sex work typology, KP programme attendance and previous or current PrEP use. DCE participants were younger and more likely to have previously attended the KP programme, have been offered PrEP and be currently taking PrEP than RDS participants from other survey sites (table 3).

**Table 3:** Sociodemographic and sexual behavioural characteristics of FSW workers reporting HIV negative status during RDS surveys, by participation in the DCE study, RDS-II weighted (N=2392)

Characteristics	HIV negative participants from all 22 sites (N=2392)	HIV negative participants 18 sites without DCE (N=1958)	HIV negative DCE participants (N=435)	P value
	n (RDS weighted %)	n (RDS weighted %)	n (RDS weighted %)	
Age				
18–24	834 (37.7%)	636 (35.4%)	198 (48.3%)	<0.001
≥25	1558 (62.3%)	1322 (64.6%)	237 (51.7%)	
Years in sex work				0.30
≤2 years	523 (24.4%)	420 (23.6%)	103 (27.9%)	
>2 years	1869 (75.6%)	1538 (76.4%)	332 (72.1%)	
Marital status				0.48
Single/never married	694 (30.3%)	558 (29.5%)	136 (33.7%)	
Married/cohabiting	156 (6.6%)	132 (6.7%)	24 (6.1%)	
Widowed/divorced	1542 (63.1%)	1268 (63.8%)	275 (60.2%)	
Education				0.36
None/primary	510 (22.4%)	409 (22.3%)	101 (22.9%)	
Secondary or higher	1878 (77.4%)	1545 (77.5%)	334 (77.1%)	
Ever attended key population programme				0.01
Yes	1491 (72.2%)	1204 (71.1%)	288 (77.1%)	
No	530 (27.8%)	455 (28.9%)	75 (22.9%)	
Condomless sex				0.84
Yes	1817 (78.0%)	1489 (78.2%)	328 (77.2%)	
No	575 (22.0%)	469 (21.8%)	107 (22.8%)	
Ever offered PrEP				0.01
Yes	794 (31.0%)	622 (30.0%)	172 (35.2%)	
No	1563 (67.4%)	130 (68.4%)	257 (63.3%)	
Ever taken PrEP				0.05
Yes	734 (92.1%)	581 (93.1%)	153 (88.5%)	
No	734 (92.1%)	41 (6.9%)	19 (11.5%)	
Currently taking PrEP				0.001
Yes	613 (24.4%)	471 (23.0%)	142 (30.6%)	
No	1744 (74.0%)	1457 (75.4%)	287 (68.0%)	
Intimate partner violence				0.19
Yes	1222 (50.4%)	988 (50.3%)	234 (51.1%)	
No	1170 (49.6%)	970 (49.7%)	201 (48.9%)	
Common mental disorder				0.75
Yes	1438 (59.7%)	1180 (59.9%)	259 (59.2%)	
No	954 (40.3%)	778 (40.1%)	176 (40.8%)	
Alcohol use disorder				0.36
Low risk	1574 (68.7%)	1301 (67.9%)	274 (68.9%)	
Medium risk	439 (16.9%)	351 (18.4%)	88 (16.5%)	
Severe risk	379 (14.4%)	306 (13.7%)	73 (14.6%)	

DCE, discrete choice experiment; FSW, female sex worker; PrEP, pre-exposure prophylaxis; RDS, respondent-driven sampling.

The values and preferences for long-acting PrEP fell into three broad constructs of the CFIR framework: product characteristics, provider and service delivery characteristics and individual characteristics.^25^

### Product characteristics

#### Opportunities

LAI PrEP was preferred over daily pills, particularly among those who had experienced poor oral PrEP adherence, including mobile sex workers and those reporting substance use or addiction. They liked the discretion LAI PrEP affords, preferring not to have to explain pill supplies or to experience stigma related to assumptions about their HIV status, partners or sexual practices. Female

I live with my parents, and they don’t know that I am a sex worker, if they see me with the pills, they will want to know what they are for. I will be forced to explain myself; I risk being beaten, chucked out of the home, labelled as a sex worker, and being found out that I do sex work. FSW, FGD1, Harare

FSWs who were taking daily hormonal contraceptives expressed reluctance to take more pills. FSWs perceived LAI PrEP as more efficacious, safer and more manageable than a daily oral regimen. FSW perceived LAI PrEP as safer and more discreet than a DVR.

I think the injection works better than the pills because we all know injections work faster than pills. FSW, FGD17, Mutare

#### Barriers

Potential barriers to uptake included difficulty adhering to frequent injection schedules, due to opportunity costs worse among highly mobile FSW. Participants wanted longer term options than 2 or 3 monthly, with 6 monthly cited as ideal.

Two months is too short a period for the injection, why can’t it last for six months or longer or vaccines please? Even the 8 weeks visits are too much. FSW, FGD3, Harare

With the DVR, FSW expressed concerns about the discomfort of inserting the ring and were worried about the risk of coinfections or even cervical cancer.

What if they[clients] feel the ring during sex? I would rather have the injection, no one will know I am taking PrEP. FSW, FGD20, Mutare

### Service delivery and provider characteristics

#### Opportunities

Across groups and ages, participants reported that targeted delivery of long-acting PrEP for sex workers would likely fuel stigma and instead should be offered across social groups, i.e. offered and delivered to anyone sexually active not just sex workers.

LA PrEP should not only target sex workers or other key populations. They should target all people. Targeting is discriminatory. FSW, FGD26, Mutare

#### Barriers

Sex workers who reported currently or ever taking PrEP primarily accessed it from the Key Population programme clinics. When they travel and/or can't access a Key Population programme clinic for refills, they opt not to attend another PrEP provider for refills. They anticipated this would be the same with long-acting PrEP. FSWs reported that having to pay for long-acting PrEP would be a barrier to use.

…if I must pay to get the injection, I will rather not take it. I hope CeSHHAR will provide the injection along with the pill and condom and family planning at the clinic or drop-in centres and for free too. FSW, FGD4, Harare

There were parallels drawn with contraception, FSWs preferring to have oral PrEP for bridging in the event of difficulty accessing long-acting PrEP on time as occurs with Depo Provera.

Distance from my house to where the LA PrEP is important. Too long is not good transport-wise, but close is good. Not in my hood though. I don’t want people to know I take that. FSW, FGD10, Harare

There were concerns about a range of health service delivery issues, including stigma from health staff, frequent power outages affecting injection quality and drug stockouts (already sometimes an issue with oral PrEP). In the event of discontinuation due to stockouts, sex workers reported reluctance to go through initiation again and were worried about the risk of developing drug resistance.

I have had challenges in the past where I would visit the clinic to get my pill refill and I would be told that there aren’t any pills at the clinic so I would then have to stop taking the pills until they were available at the clinic again. Won't the same thing happen with the injection? FSW, FGD4, Harare

FSW did not want to access PrEP through HIV testing or treatment services in case need for PrEP was confused with need for HIV treatment. In addition, FSW reported concerns about the need to provide personal data to access PrEP, expressing fears of being targeted by police, the military or abused by community members. FSW wanted to limit the role of peer educators to providing information and adherence support and did not want to access PrEP through them.

### Individual characteristics

#### Opportunities

FSWs across all ages said they wanted to have access to LAI PrEP. A few sex workers would prefer to use free condoms to protect themselves rather than pay money to travel to get PrEP and risk exposing their engagement in sex work.

#### Barriers

Sex workers experiencing poor mental health, substance use, homelessness and poverty were concerned about developing drug resistance as they anticipated that they would inevitably have dosing interruptions. Fear of side effects (preinitiation) or experience of side effects (postinitiation) was cited as a barrier to uptake and PrEP continuation and was a concern about how to stop LAI PrEP safely.

I don’t like needles so I don’t think I will take the injection. FSW, FGD6, Harare

Are there any side effects from taking the injection for too long, I mean can I develop some drug resistance or develop some lipodystrophy, or chronic fatigue or muscle loss? FSW, FGD 28, Mutare

### The RDS survey

We enrolled 4444 FSWs, of whom 54.1% (n=2392) reported HIV negative status. A number of coupons were distributed and returned, and RDS diagnostics for each site, including recruitment trees, combined convergence and bottleneck plots for the primary outcomes have been reported in detail elsewhere.^17^ Overall, 37.7% (n=834/2392) were <25 years, 77.4% (n=1878/2392) had reached secondary school. The majority (n=2032/2392, 84.6%) reported they would want to take PrEP every day if it almost completely reduced their chances of contracting HIV, and 24.8% (603/2392) reported that they were currently taking oral PrEP.

In terms of choice of long-acting PrEP options (Question: There are new PrEP options that will become available in the next year. Which of these would you be interested in taking? 1835/2392 (74.1%) indicated that they would be interested in using LAI PrEP, 230/2392 (10.6%) the DVR, 59/2392 (2.4%) either of the two options, and 268/2392 (12.9%) neither of the two options. Overall, when asked to state a preference of one option over another, 1966/2932 (80.7%) stated they preferred LAI PrEP over daily oral PrEP, 984/2392 (42.2%) preferred the DVR over daily oral PrEP, and 1809/2392 (74.1%) stated they preferred LAI PrEP over the DVR. The results were consistent over age and geography for DCE sites (see online supplemental appendix table S2).

### Discrete choice experiment

**Figure 2:**
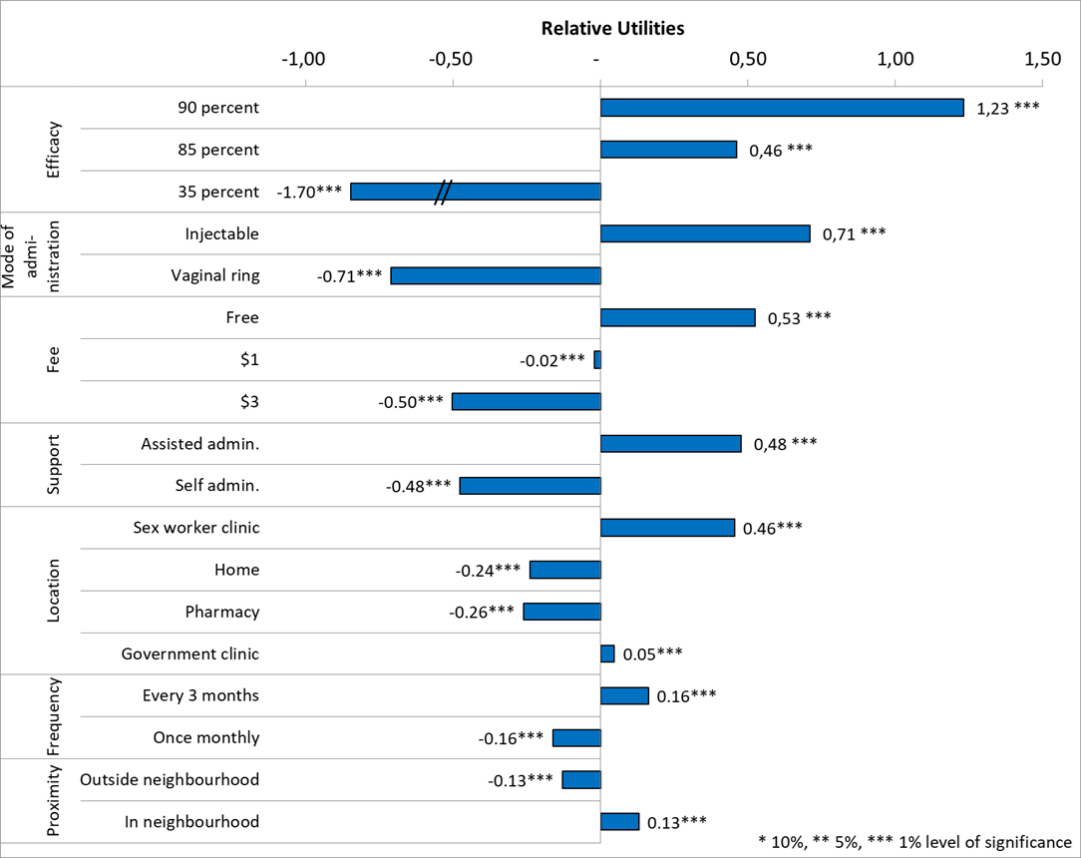
Random parameter logit—main effects (model 3). *10%,**5%, *** 1% level of significance.

The strongest relative preference was for long-acting PrEP efficacy, where 90% efficacy was associated with a utility U=1.233, p<0.01 (figure 2). Participants strongly preferred LAI PrEP (U=0.711, p<0.01) over the vaginal ring. Long-acting PrEP price was also a strong driver of preferences with free options associated with a utility U=0.525, p<0.01. As strongly, participants preferred assisted long-acting PrEP administration (U=0.477, p<0.01) over self-administered long-acting PrEP. For location to access long-acting PrEP, sex worker clinics were the most preferred (U=0.455, p<0.01) followed by government clinics (U=0.455+(–0.262 to 0.240)=0.047, p<0.01), over pharmacy (U=−0.262, p<0.01) and home (U=−0.240, p<0.01). To a lesser extent, sex workers preferred to receive their long-acting PrEP option in their neighbourhood (U=0.130, p<0.01), once every 3 months (U=0.162, p<0.01), rather than in the neighbourhood, monthly.

While most participants showed a strong preference to adopting a long-acting PrEP option rather than opting out (U=−3.726, p<0.01), the opt-out was more often chosen among those who identified as irregular testers (U=−3.726+0.664=−3.062, p<0.10) or were above 25 years old (U=−3.726+0.496=−3.230, p<0.10). The complete model outputs are provided in the online supplemental appendix table S3.

Triangulating findings on preferences across research methods suggested that responses were consistent (table 4).

**Table 4:** Key findings on preferences and triangulation of methods

Preferences by domain	Key qualitative results (15 FGDs) (N=102)	DCE results (N=435)	RDS results (N=2392)	Triangulation results
Product characteristics	Preference for LAI PrEP over DVR and oral tablets—adherence, privacy, efficacy. Longer interval between injections preferred.	Strong preference for LAI PrEP over DVR; high efficacy was critical. Less frequent injections were preferred.	Strong preference for LAI over DVR or daily oral PrEP	Consistent
Service and provider characteristics	Did not want targeted to sex workers. Wanted to collect from sex worker programme	Preferred to collect from sex worker programme clinics, and to a lesser extent within their neighbourhood.	N/A	Complementary
Individual characteristics	All ages and typologies preferred LAI PrEP	All ages preferred LAI PrEP although never tested and those aged >25 were less likely to prefer LAI PrEP than others	All ages and typologies preferred LAI PrEP (see online supplemental appendix tables S2a and S2b)	Consistent

DCE, discrete choice experiment; DVR, dapivirine vaginal ring; FGDs, focus group discussions; LAI, long-acting injectable; N/A, not applicable; PrEP, pre-exposure prophylaxis.

## Discussion

This is one of the first studies to explore values and preferences of FSW in southern Africa for long-acting PrEP. We found that there was strong interest in long-acting PrEP among Zimbabwean FSW, highlighting the potential for these products to broaden choice. We found strong concordance across research approaches with most FSW indicating that they preferred the option of LAI PrEP to both daily oral PrEP and DVR. There was no variation in preferences by age or geography (see online supplemental appendix table S2). The DVR was only preferred by a minority. LAI PrEP addressed privacy and adherence concerns to some extent, although FSWs felt that 6-monthly injections would be preferable to less frequent injections because of their high rates of mobility. Of note, this study was conducted in 2021 before the results of the PURPOSE 1 lenacapavir trial.^14^ FSW wanted LAI PrEP to be offered across social groups not just to key populations, which they envisaged would cause the product to become stigmatised. There was a lack of trust in healthcare providers in the public sector and low interest in pharmacy delivery, with the majority opting to access PrEP through programmes specifically designed for sex workers, with some stating they would miss the opportunity for refill pick up/repeat injection if away from home and the public sector was the only available option. Issues of privacy (related to PrEP and being a sex worker), confidentiality and respect emerged as key qualitative themes.

The strengths of this study are its use of qualitative and quantitative methods to elicit values and preferences in addition to formally determining the relative preferences of FSW in a rigorously designed and conducted DCE. The study was nested within Zimbabwe’s nationally scaled Key Populations programme for sex workers^18^ and within the endline population-based survey for a cluster randomised trial which recruited over 4400 FSW from across 22 sites in Zimbabwe using RDS, the UNAIDS recommended approach for recruiting representative samples of hidden populations. RDS diagnostics^17^ (data not shown) did not show evidence of biased recruitment. Research participants included FSW of varying age, educational level, sex work typology, past programme exposure/clinic attendance and past/current PrEP use.

Limitations of the study are that DCEs measure only the hypothetical acceptability of product features rather than preferences after using actual products (although a proportion of participants had tried daily oral PrEP). We also didn’t include questions about the hypothetical acceptability of long-acting tablets in any aspects of the research as these were not considered an imminent possibility. Similarly, there was no pharmacy-delivered PrEP available in Zimbabwe, so this was also a hypothetical scenario to assess. second, research exploring the external validity of DCEs has suggested positive predictive values to be reasonably high in accurately predicting choices; in contrast, negative predictive values have been more moderate.^32 33^ Of note, the preferences and trade-offs evaluated are based only on those attributes included in the design. We tried to address this by focusing on modifiable attributes identified through extensive formative research. Also, including an opt-out alternative in the DCE allowed us to measure actual demand for the long-acting PrEP products. While CAB LA is administered every 2 months and lenacapavir every 6 months, we reduced the number of levels for this attribute (from 4 levels: 1/2/4/6 months, to 2 levels: 1/3 months) to increase the DCE design efficiency and reduce the number of DCE participants required. It would have been useful to have compared 2 monthly with 6 monthly administration to allow more direct comparison of CAB LA and lenacapavir. We acknowledge that oral PrEP, but not LAI PrEP, is available 3 monthly and accept this as a limitation of our findings.

A recent systematic review of values and preferences for LAI PrEP included mainly qualitative studies with few FSW from Africa.^15^ There were few DCEs. The findings were similar to those found here. There was an overall interest in and often a preference for LAI PrEP. Many stakeholders indicated that LAI PrEP could help address adherence challenges associated with daily or on‐demand dosing for oral PrEP and may be a better lifestyle fit for individuals seeking privacy, discretion and infrequent dosing.

Understanding values and preferences among FSW from Africa for long-acting PrEP options can be used to inform design of implementation approaches as well as strategies to optimise uptake and continuation. Our data suggest that having a choice of PrEP options has the potential to motivate people who would not otherwise take PrEP to consider doing so, which builds on the Dynamic Choice trials from Kenya and Uganda, which show that offering a choice of biomedical prevention results in higher rates of prevention coverage.^34 35^ We found keen interest in long-acting PrEP among FSW, highlighting the potential for these products to broaden choice. Sex workers valued culturally appropriate and affirming care provided by competent healthcare workers in a welcoming environment free of discriminatory attitudes—and preferred accessing long-acting PrEP through programmes specifically catering to the needs of sex workers run by appropriately trained healthcare workers. These services are under threat because of resource constraints which will likely be a false economy. As HIV epidemics become better controlled across Africa, the relative importance of transmission associated with sex work will likely increase with the potential for repeated outbreaks of HIV if interventions to keep sex workers healthy reduce in intensity.^36^ Modelling suggests that increasing the intensity of programmes for FSW in the region would offer cost-effective and beneficial impact on population incidence.^37^

FSWs did not want to pay for long-acting PrEP options with cost being a strong predictor of potential uptake. As resources for HIV become increasingly constrained, there may be pressure to integrate services for sex workers within broader public health services, and indeed this is already being advocated by major donors. Our data suggest this would likely compromise PrEP uptake and continuation among FSW. Services targeting FSW in Africa are likely to be highly cost-effective and modelling suggests they should likely be intensified rather than scaled back.^37 38^ FSWs expressed concerns about developing drug resistance to LAI-PrEP in the event that they were unable to adhere, a legitimate concern for CAB LA where poor adherence could induce cross-resistance with dolutegravir, a first line drug for treatment in much of Africa. Lenacapavir is a capsid inhibitor and the first in its class currently approved for use, so cross-resistance is currently less of an issue. Interventions to support adherence and ensure users are aware of the trade-offs between the two drugs will be important as they are rolled out.

Introducing resources to address mental health, alcohol and structural factors in existing KP-friendly and supportive environments was considered important. FSW generally perceived both CAB-LA and DVR to address stigma and thought their availability would facilitate PrEP uptake, although they were worried that the high frequency of injection schedules for CAB LA would be burdensome, a concern that would likely be addressed with scale up on lenacapavir.

Administering long-acting PrEP at alternate locations other than the conventional HIV care settings, such as KP drop-in centres and pharmacies, could also be helpful, although few FSWs preferred pharmacies as a delivery venue, possibly because they had no actual experience of pharmacy-delivered services. Participants preferred assisted long-acting PrEP administration, within specialist sex worker services, suggesting that they value a delivery channel they have heard of; 66% (288/435) of DCE participants had ever attended a KP programme clinic. FSWs are concerned about the preparedness of the health delivery system to address delivery barriers around cost, staff capacity and ensuring continuity of supply. Addressing staff capacity needs will require training to ensure concerns around discriminatory behaviour are addressed. Additional analyses (see online supplemental appendix table S2) aimed to assess whether preferences varied between sociodemographic groups including geography and sex work typology, but we did not find sufficient difference to suggest that long-acting PrEP service delivery channels would need to take these into account. Although irregular testers (not tested in the last 12 months) and those ≥25 years are slightly more likely to opt out rather than choose a long-acting PrEP option compared with regular testers and those <25 years old respectively, they are still strongly preferring to opt for a long-acting PrEP than to opt out.

These study findings offer new perspectives on the values and preferences of FSW, as well as likely barriers and opportunities to support long-acting PrEP uptake across FSW of different ages and typologies in Zimbabwe.

## Supplementary material

10.1136/bmjgh-2025-021333 online supplemental file 1

10.1136/bmjgh-2025-021333 online supplemental file 2

## Data Availability

Data are available on reasonable request.
