# Supplementary material for: Values and preferences of female sex workers in Zimbabwe for long-acting injectable pre-exposure prophylaxis and the dapivirine vaginal ring: results of a mixed-methods research study
Source: BMJ Glob Health. 2026 Mar 2;11(3):e021333. doi: 10.1136/bmjgh-2025-021333 (PMC12958915; doi:10.1136/bmjgh-2025-021333)
Supplement: online supplemental file 1 [file bmjgh-11-3-s001.docx]

**Supplementary appendix for**

**Values and preferences of female sex workers in Zimbabwe for long-acting injectable pre-exposure prophylaxis and the dapivirine vaginal ring: results of a mixed methods research study**

**Appendix**

Table S1. Questions on long-acting PrEP included in the RDS survey questionnaire
Table S2(a). Pre-exposure prophylaxis preferences by age
Table S2(b). Pre-exposure prophylaxis preferences by site
Table S3. Estimation of preferences for LA-PrEP options among sex workers by age group and HIV testing history
Table S4. Example DCE Choice set

**Table S1. Questions on long-acting PrEP included in the RDS survey questionnaire**

| **Questions** | **Responses** |
| --- | --- |
| 1. There are new PrEP options that will become available in the next year. Which of these would you be interested in taking? | • Long-acting Injectable PrEP (this is an injection that a woman who does not have HIV gets once every 2 months to prevent HIV infection)  • Long-acting Dapivirine Ring (this is a ring that a woman inserts into her vagina once every month. The ring stays in place throughout the month releasing a small dose of a drug to prevent HIV.  • None of the above |
| 2. Would having long-acting injectable PrEP available that you had to take every 2 months make it easier for you to take up PrEP than taking a daily tablet? | • No  • Yes |
| 3. Would having long-acting PrEP available in a vaginal ring that you have to change every month make it easier for you to take up PrEP than taking a daily tablet? | • No  • Yes  • Not applicable |
| 4. Would having long-acting injectable PrEP available that you had to take every 2 months make it easier for you to take up PrEP than having long-acting PrEP available in a vaginal ring that you have to change every month? | • No  • Yes  • Not applicable |

|  | **18-24 years**  **(N=196)**  **n/N (%)** | **25+ years**  **(N=233)**  **n/N (%)** | **p-value** |
| --- | --- | --- | --- |
| **There are new PrEP options that will become available in the next year. Which of these would you be interested in taking?** |  |  |  |
| Long-acting Injectable PrEP only | 143/196 (69.5) | 187/133 (75.8) | 0.23^†^ |
| Long-acting Dapivirine Ring only | 21/196 (11.9) | 23/133 (11.1) |  |
| Long-acting Injectable PrEP or Dapivirine Ring | 5/196 (3.0) | 4/133 (2.1) |  |
| None | 27/196 (15.6) | 19/133 (11.0) |  |

**Table S2(a). Pre-exposure prophylaxis preferences by age**

PrEP, pre-exposure prophylaxis
^†^Fisher’s exact p-value

**Table S2(b). Pre-exposure prophylaxis preferences by site**

|  | **Site 1**  **(N=96)**  **n/N (%)** | **Site 2**  **(N=108)**  **n/N (%)** | **Site 3**  **(N=122)**  **n/N (%)** | **Site 4**  **(N=103)**  **n/N (%)** | **p-value** |
| --- | --- | --- | --- | --- | --- |
| **There are new PrEP options that will become available in the next year. Which of these would you be interested in taking?** |  |  |  |  |  |
| Long-acting Injectable PrEP only | 73/96 (72.0%) | 87/108 (73.6%) | 97/122 (75.3) | 73/103 (69.6) | 0.64^†^ |
| Long-acting Dapivirine Ring only | 12/96 (12.2%) | 10/108 (13.0%) | 12/122 (10.9) | 10/103 (9.5) |  |
| Long-acting Injectable PrEP or Dapivirine Ring | 2/96 (3.3%) | 2/108 (2.1%) | 3/122 (3.1) | 2/103 (1.7) |  |
| None | 9/96 (12.5%) | 9/108 (11.3%) | 10/122 (10.7) | 18/103 (19.2) |  |

PrEP, pre-exposure prophylaxis
^†^Fisher’s exact p-value

**Table S3. Estimation of preferences for LA-PrEP options among sex workers by age group and HIV testing history**

|  | **Model 1 (multinomial logit)** | | **Model 2 (nested logit)** | | **Model 3 (random parameter logit)** | | | |
| --- | --- | --- | --- | --- | --- | --- | --- | --- |
| Attributes | **ß** | **SE** | **ß** | **SE** | **ß** | **SE** | **SD** | **SE** |
| **Main effects** |  |  |  |  |  |  |  |  |
| Location (government clinic) |  |  |  |  |  |  |  |  |
| Pharmacy | -0,153*** | 0,032 | -0,157*** | 0,033 | -0,262*** | 0,058 | 0,212** | 0,104 |
| Sex worker clinic | 0,145*** | 0,033 | 0,184*** | 0,044 | 0,455*** | 0,067 | 0,491*** | 0,082 |
| Home | -0,124*** | 0,045 | -0,100** | 0,049 | -0,240*** | 0,074 | 0,003 | 0,134 |
| Proximity (in my neighbourhood) |  |  |  |  |  |  |  |  |
| Outside my neighbourhood | -0,092*** | 0,019 | -0,082*** | 0,020 | -0,130*** | 0,034 | 0,259*** | 0,049 |
| Frequency (once monthly) |  |  |  |  |  |  |  |  |
| Once every 3 months | 0,081*** | 0,016 | 0,085*** | 0,017 | 0,162*** | 0,031 | 0,234*** | 0,063 |
| Fee ($1) |  |  |  |  |  |  |  |  |
| Free | 0,251*** | 0,051 | 0,263*** | 0,053 | 0,525*** | 0,082 | 0,525*** | 0,082 |
| $3 | -0,178*** | 0,029 | -0,223*** | 0,043 | -0,503*** | 0,058 | 0,503*** | 0,058 |
| Efficacy (35%) |  |  |  |  |  |  |  |  |
| 85% | 0,096*** | 0,036 | 0,185** | 0,074 | 0,462*** | 0,081 | 0,519*** | 0,093 |
| 90% | 0,538*** | 0,030 | 0,610*** | 0,059 | 1,233*** | 0,090 | 1,109*** | 0,080 |
| Mode of administration (vaginal ring) |  |  |  |  |  |  |  |  |
| Injection | 0,300*** | 0,021 | 0,341*** | 0,036 | 0,711*** | 0,063 | 0,842*** | 0,074 |
| Support (self-administration) |  |  |  |  |  |  |  |  |
| Assisted administration | 0,223*** | 0,039 | 0,253*** | 0,045 | 0,477*** | 0,097 | 0,378*** | 0,049 |
| Neither (none of the LA-PrEP options) | -0,903*** | 0,081 | -1,104*** | 0,135 | -3,726*** | 0,496 | 4,988*** | 0,353 |
| **Interaction effects** |  |  |  |  |  |  |  |  |
| Neither x Older | 0,233*** | 0,044 | 0,232*** | 0,044 | 0,496* | 0,272 | 1,818*** | 0,233 |
| Neither x Irregular testers | 0,360*** | 0,079 | 0,355*** | 0,079 | 0,664* | 0,401 | 1,734*** | 0,207 |
| Sex worker clinic x Older | 0,013 | 0,025 | 0,010 | 0,025 | 0,045 | 0,047 | 0,446*** | 0,071 |
| Once every 3 months x Older | -0,027* | 0,016 | -0,028* | 0,016 | -0,050* | 0,030 | 0,065 | 0,096 |
| $3 x Older | 0,058** | 0,023 | 0,057** | 0,023 | 0,129*** | 0,043 | 0,129*** | 0,043 |
| Injection x Older | -0,030* | 0,017 | -0,029* | 0,017 | -0,091 | 0,056 | 0,874*** | 0,075 |
| Free x Irregular testers | 0,051 | 0,049 | 0,051 | 0,051 | 0,070 | 0,077 | 0,028 | 0,090 |
| Assisted-administration x Irregular testers | 0,148*** | 0,038 | 0,153*** | 0,039 | 0,280*** | 0,095 | 0,255*** | 0,056 |
| **Model fit statistics** |  |  |  |  |  |  |  |  |
| Number of participants | 435 |  | 435 |  | 435 |  |  |  |
| Number of observations | 4956 |  | 4956 |  | 4956 |  |  |  |
| AIC | 9105.6 |  | 9105.3 |  | 6468.7 |  |  |  |
| AIC/N | 1.837 |  | 1.837 |  | 1.305 |  |  |  |
| IV parameter (nested logit) |  |  | 0,741*** |  |  |  |  |  |

*10%, **5%, ***1% level of significance with p value
AIC, Akaike information criterion; SD, standard deviation; SE, standard error
Since effects coding was applied, within each attribute, utility coefficients add up to zero, that is for two-level attributes, the coefficient of the omitted level is the same magnitude with opposite sign

**Table S4. Example DCE Choice set**

|  | **CODE** | **ATTRIBUTE** | **DEFINITION** | **LEVELS** | | | | | | |
| --- | --- | --- | --- | --- | --- | --- | --- | --- | --- | --- |
| 1 | Location | Location where the product is available | A place where you get the product | **PHARMACY** | | **SEX WORKER CLINIC** | | **HOME DELIVERED** | | **GOVERNMENT CLINIC** |
|  |  |  |  | 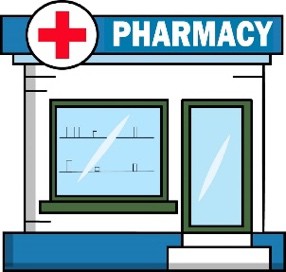 | | 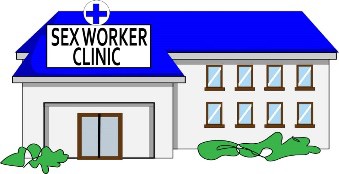 | | 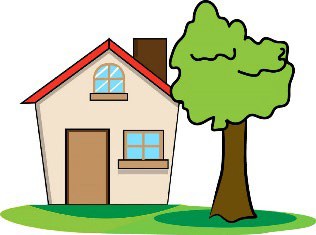 | | 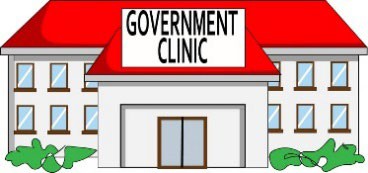 |
| 2. | Proximity | Proximity of facility to get product | Nearness of a health facility from where you live | **OUTSIDE MY NEIGHBOURHOOD** | | | | **IN MY NEIGHBOURHOOD** | | |
|  |  |  |  | 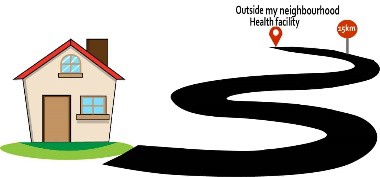 | | | | 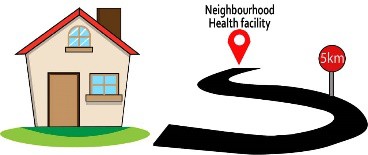 | | |
| 3. | Mode | Mode of administration | The way in which the PrEP option is delivered for use | **VAGINAL RING** | | | | **INJECTION** | | |
|  |  |  |  | 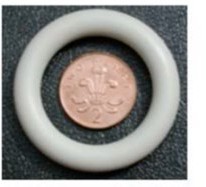 | | | | 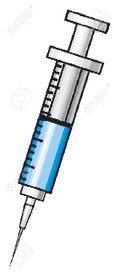 | | |
| 4. | Support | Support for administering the product | The way of having the PrEP option in your body | **SELF-ADMINISTRATION** | | | | **ASSISTED ADMINISTRATION** | | |
|  |  |  |  | 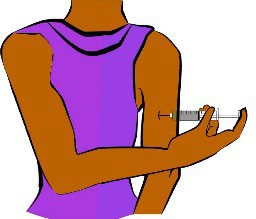 | 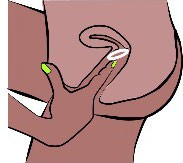 | | | 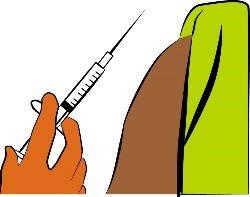 | | 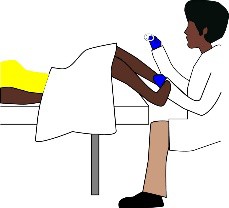 |
| 5. | Frequency | Frequency of receiving the product | How often you need to receive or take the PrEP option | **EVERY MONTH** | | | | **EVERY THREE MONTHS** | | |
|  |  |  |  | 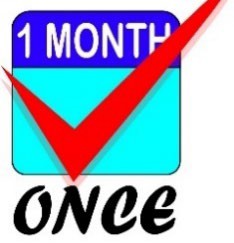 | | | | 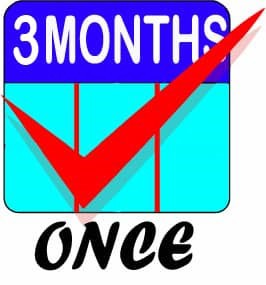 | | |
| 6. | User fee | User fee for the product | The amount of money you are willing to pay for the PrEP option | **$0** | | | **$1** | | **$3** | |
|  |  |  |  | 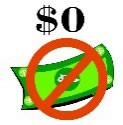 | | | 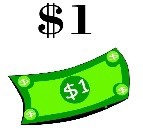 | | 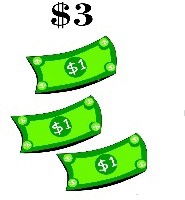 | |
| 7. | Efficacy | Product efficacy | The level of effectiveness in reducing the risk of acquiring HIV | **35%** | | | **85%** | | **90%** | |
|  |  |  |  | 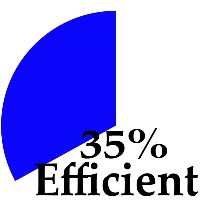 | | | 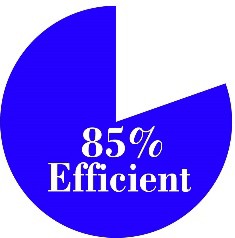 | | 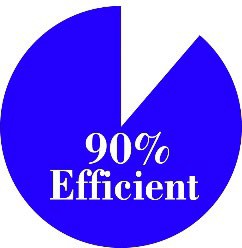 | |
