# Supplementary material for: Values and preferences of female sex workers in Zimbabwe for long-acting injectable pre-exposure prophylaxis and the dapivirine vaginal ring: results of a mixed-methods research study
Source: BMJ Glob Health. 2026 Mar 2;11(3):e021333. doi: 10.1136/bmjgh-2025-021333 (PMC12958915; doi:10.1136/bmjgh-2025-021333)
Supplement: online supplemental file 2 [file bmjgh-11-3-s002.docx]

### BMJ Global Health Author Reflexivity Statement

Adapted from Morton, B., Vercueil, A., Masekela, R., Heinz, E., Reimer, L., Saleh, S., Kalinga, C., Seekles, M., Biccard, B., Chakaya, J., Abimbola, S., Obasi, A. and Oriyo, N. (2022), Consensus statement on measures to promote equitable authorship in the publication of research from international partnerships. Anaesthesia, 77: 264-276. <https://doi.org/10.1111/anae.15597>

| **Study conceptualisation** | |
| --- | --- |
| 1. How does this study address local research and policy priorities? | LAI PrEP is being scaled up in Zimbabwe and the southern African region more generally and these results have been important in informing policy and implementation. |
| 1. How were local researchers involved in study design? | Local researchers drafted protocol including data collection tools, submitted protocol for ethics approval, implemented the study, conducted most of the analysis (DCE analysis is was led by MD) and wrote the first draft of the manuscript. |
| **Research management** | |
| 1. How has funding been used to support the local research team(s)? | The study was nested within a Wellcome Trust collaborative award which provided extensive training for researchers in Zimbabwe, Malawi and South Africa including supporting 4 PhD students |
| **Data acquisition and analysis** | |
| 1. How are research staff who conducted data collection acknowledged? | All are co-authors |
| 1. How have members of the research partnership been provided with access to study data? |  |
| 1. How were data used to develop analytical skills within the partnership? | All RDS and qualitative analysis was done by Zimbabwean analysts. The DCE analysis was done in partnership between Dr D’Elbee and Zimbabwean analysts. |
| **Data interpretation** | |
| 1. How have research partners collaborated in interpreting study data? | All research partners contributed to interpreting study data. |
| **Drafting and revising for intellectual content** | |
| 1. How were research partners supported to develop writing skills? | First drafts were written by FM, TK (quantitative, JM, SC (survey analysis), and MD (DCE). All authors contributed to subsequent drafts. |
| 1. How will research products be shared to address local needs? | Long acting injectable PrEP is being scaled up across Zimbabwe and these findings have been taken into account by MoHCC in Zimbabwe. |
| **Authorship** | |
| 1. How is the leadership, contribution and ownership of this work by LMIC researchers recognised within the authorship? | The first author is Dr Machingura – co first author with Dr D’Elbee. |
| 1. How have early career researchers across the partnership been included within the authorship team? | Tatenda Kujeke and Jasper Munjoma are both early career researchers. SC is a mid career researcher. FM, MD, FMC are senior researchers. MR and RB are policy partners. |
| 1. How has gender balance been addressed within the authorship? | Five of eight authors are women |
| **Training** | |
| 1. How has the project contributed to training of LMIC researchers? | The study was nested within a Wellcome Trust collaborative award which provided extensive training for researchers in Zimbabwe, Malawi and South Africa including supporting 4 PhD students |
| **Infrastructure** | |
| 1. How has the project contributed to improvements in local infrastructure? | The funding from WHO specifically for this study was minimal – so it has not contributed to infrastructure development. |
| **Governance** | |
| 1. What safeguarding procedures were used to protect local study participants and researchers? | The local research partner was CeSHHAR Zimbabwe who have safeguarding policies in place which are actively implemented. |
